# SARS-CoV-2-Induced Type I Interferon Signaling Dysregulation in Olfactory Networks Implications for Alzheimer’s Disease

**DOI:** 10.3390/cimb46050277

**Published:** 2024-05-10

**Authors:** George D. Vavougios, Theodoros Mavridis, Triantafyllos Doskas, Olga Papaggeli, Pelagia Foka, Georgios Hadjigeorgiou

**Affiliations:** 1 Department of Neurology, Medical School, University of Cyprus, Nicosia 1678, Cyprus; 2 Department of Neurology, Tallaght University Hospital (TUH)/The Adelaide and Meath Hospital, Dublin, Incorporating the National Children’s Hospital (AMNCH), D24 NR0A Dublin, Ireland; mavridismdr@gmail.com; 3 Department of Neurology, Athens Naval Hospital, 115 21 Athens, Greece; doskastr@gmail.com; 4 Molecular Virology Laboratory, Hellenic Pasteur Institute, 115 21 Athens, Greece; o.papaggeli@pasteur.gr (O.P.); pfoka@pasteur.gr (P.F.)

**Keywords:** COVID-19, long COVID, Alzheimer’s disease, cognitive impairment, IFITM3, nasal epithelial cells, type I interferon signaling, cGAS-STING

## Abstract

Type I interferon signaling (IFN-I) perturbations are major drivers of COVID-19. Dysregulated IFN-I in the brain, however, has been linked to both reduced cognitive resilience and neurodegenerative diseases such as Alzheimer’s. Previous works from our group have proposed a model where peripheral induction of IFN-I may be relayed to the CNS, even in the absence of fulminant infection. The aim of our study was to identify significantly enriched IFN-I signatures and genes along the transolfactory route, utilizing published datasets of the nasal mucosa and olfactory bulb amygdala transcriptomes of COVID-19 patients. We furthermore sought to identify these IFN-I signature gene networks associated with Alzheimer’s disease pathology and risk. Gene expression data involving the nasal epithelium, olfactory bulb, and amygdala of COVID-19 patients and transcriptomic data from Alzheimer’s disease patients were scrutinized for enriched Type I interferon pathways. Gene set enrichment analyses and gene–Venn approaches were used to determine genes in IFN-I enriched signatures. The Agora web resource was used to identify genes in IFN-I signatures associated with Alzheimer’s disease risk based on its aggregated multi-omic data. For all analyses, false discovery rates (FDR) <0.05 were considered statistically significant. Pathways associated with type I interferon signaling were found in all samples tested. Each type I interferon signature was enriched by IFITM and OAS family genes. A 14-gene signature was associated with COVID-19 CNS and the response to Alzheimer’s disease pathology, whereas nine genes were associated with increased risk for Alzheimer’s disease based on Agora. Our study provides further support to a type I interferon signaling dysregulation along the extended olfactory network as reconstructed herein, ranging from the nasal epithelium and extending to the amygdala. We furthermore identify the 14 genes implicated in this dysregulated pathway with Alzheimer’s disease pathology, among which HLA-C, HLA-B, HLA-A, PSMB8, IFITM3, HLA-E, IFITM1, OAS2, and MX1 as genes with associated conferring increased risk for the latter. Further research into its druggability by IFNb therapeutics may be warranted.

## 1. Introduction

Beyond its acute phase, COVID-19 has been associated with a wide range of neuropsychiatric sequelae and, perhaps most prominently, cognitive impairment. Though initially underrecognized, COVID-19’s cognitive consequences appear to develop on a biological substrate determined using the aftermath of host–virus interactions [1]. 

In a previous study, we hypothesized that the biological substrate of COVID-19’s effects on cognition involves the induction of type I interferon signaling peripheral to the central nervous system, such as the olfactory epithelium, and subsequently its propagation centrally to functionally connected sites such as the limbic system [2]. We furthermore identified this innate immune pathway as shared between COVID-19 transcriptomes and those extracted from the entorhinal cortex of Alzheimer’s disease patients. While our initial hypothesis was explored on the premise of limited available data, several subsequent independent studies since then have provided further support to our model (some of which were reviewed in [3,4]) and even reiterated the idea of a type I interferon-centric insult to the CNS following exposure to SARS-CoV-2 [5]. 

The attractiveness of type I interferon signaling as the putative mechanism for COVID-19’s neuropsychiatric consequences relies on its mechanistic relationships with innate immunity and cognition, as well as its consistent challenge by SARS-CoV-2. Specifically, type I interferon (IFN-I) signaling is the premier antimicrobial pathway mobilized in response to a pathogen or a pathogen-associated molecular pattern (PAMP), contested by SARS-CoV-2 and potentially shaping the severity and consequences of COVID-19. Type I interferon signaling has both direct effects on cognition by impairing neuronal plasticity [6] and long-term effects such as the impairment of neo-neurogenesis [7], a critical function in the olfactory bulb and the hippocampus, mediating olfactory processing and cognition, correspondingly [8]. In our model, we examined whether type I interferon signaling dysregulation and, specifically, cascades involving the interferon-inducible protein 3 (IFITM3) were common between SARS-CoV-2 infection and Alzheimer’s disease. Another important consideration is that type I interferon signaling is ubiquitous across cells, and tonic IFN-I regulates peripheral innate immune responses, canonical/housekeeping microglial states in the CNS, and the crosstalk between the periphery and the CNS [9].

The crosstalk between peripheral and central type I interferon responses belies the intended comparison between SARS-CoV-2 as an IFN-I stimulant and the dysregulations in type I interferon signaling evident in Alzheimer’s disease. At least one mechanism involving IFN-I in Alzheimer’s disease pathobiology involves its upregulation in microglia primed by neurofibrillary tangles and adjunct nucleic acids, regardless of their intrinsic or xenobiotic origin; the consequences of this upregulation were shown to drive neuroinflammation and lead to synapse loss [10]. Another potentially important mechanism by which IFN-I contributes to Alzheimer’s disease mechanisms is the disruption of blood-brain-barrier (BBB) integrity, where upregulated IFN-I cascades have been observed in brain endothelial cells [11]. Another crucial junction between IFN-I dysregulation and Alzheimer’s disease pathobiology occurs via the second messenger cyclic GMP–AMP (cGAS)-cyclic GMP–AMP receptor stimulator of interferon genes (STING) (cGAS-STING) pathway directly, which upregulates IFN-I signaling as a response to the detection of cytosolic DNA [12]. Recently, pathogenic tau was shown to activate cGAS-STING and consequently upregulate IFN-I responses in microglia in a murine model of Alzheimer’s disease [13]. In a cellular model of neuroinflammation, Aβ treatment was shown to upregulate cGAS-STING and regulate IFITM3, an antiviral protein that modulates gamma-secretase activity [14]. IFITM3 is another notable junction where innate immunity encounters neuroinflammation and Alzheimer’s disease mechanisms. IFITM3 functions as an antiviral protein that may be subverted by SARS-CoV-2 and enhance rather than restrict infection [15,16]. Furthermore, several studies, including those from our group have outlined IFITM3 pathways as overlapping networks between Alzheimer’s disease and COVID-19 [2,17,18,19,20,21]. IFITM3 was recently shown to modulate amyloidogenic APP processing by gamma secretase [14], and partake in IFN-I responsive circuits following microglial uptake of Aβ and Aβ–Nucleic Acid complexes [10]. 

Taken together, these studies indicate that dysregulations in IFN-I may affect molecular pathways critical to its pathogenesis, such as beta-amyloid production, tauopathy, and microglial polarization. As a syndrome, impaired cognition has been identified within the spectrum of COVID-19’s post-acute syndromes [1,22] and is associated with impaired olfaction [23]. Notably, impaired odor identification has also been correlated with neurodegeneration in the setting of Alzheimer’s disease [24] and has shown potential as a preclinical marker [25]. Considering that neurodegeneration in the extended olfactory network and its connected areas has been both previously proposed [26] and identified [27] in COVID-19 survivors, the question arises of whether IFN-I perturbations would also be present in the transcriptomes of the extended olfactory network regions.

Supporting this hypothesis, IFN-I pathways enriched by IFITMs have re-emerged both in nasal epithelia [28,29] and the frontal lobe of COVID-19 patients [30] as part of the innate immune response and consequent activation of interferon-stimulated gene networks. Dysregulated IFITM3 networks in brain endothelial cells in the setting of COVID-19 have been previously identified [20], providing further support to our hypothesis of an outside-in quasinfectious transmission of this dysregulation to the CNS [2]. 

We therefore sought to replicate our model of an outside-in transmission of IFN-I signaling via the transolfactory route, the pathway by which SARS-CoV-2 may gain entry into the CNS [31]. To do so, we aimed to identify significantly enriched IFN-I signatures and genes along the transolfactory route employing published datasets of the nasal mucosa and olfactory bulb amygdala transcriptomes of COVID-19 patients and determine the presence of IFITM3 specifically in those gene networks. Furthermore, we aimed to determine whether signatures and genes detected in COVID-19 transcriptomes were also present in transcriptomes from CNS tissue donated by Alzheimer’s disease patients and associated with increased risk.

## 2. Materials and Methods

### 2.1. Concept Design

In this study, we attempt a hypothesis-driven analysis that focuses on the amygdala as a hub for monosynaptic transmission and, hence, a site potentially vulnerable to other cell-to-cell signals, including IFN signaling. Our conceptual design furthermore considers that IFN-I is the canonical, if later deregulated, response to SARS-CoV-2, and one that may be propagated from an immune-challenged cell to other non-immune challenged cells [32]. Therefore, we specifically consider genes enriching IFN-I in that setting and consider IFN-I signatures in tissues that are anatomically and functionally connected: the olfactory epithelium, the olfactory bulb, and the amygdala. For the nasal epithelial cells, the ciliated cell subset was selected as the prime target of SARS-CoV-2 and a site of active replication [33]. For our reconstruction of this hypothetical pathway, we, therefore, consider a site of primary infection and its neuroanatomical correlates up until the amygdala, selecting genes enriching the IFN-I interferon signature with a specific interest in IFITM- and OAS- family genes.

In our original hypothesis, the IFN-I response against endogenous DAMPs such as Aβ would be enhanced by an exogenous IFN-I signal [3]. In order to determine the overlap between these signatures and those previously reported in Alzheimer’s disease transcriptomes, we specifically selected the study of Das and colleagues [34] as it provides spatially resolved transcriptomic data on IFN-I responses elicited by Aβ plaques. Shared genes between IFN-I responses in the nasal epithelium (ciliated cells), OB, Amygdala, and the IFN-I response versus amyloid plaques would then be scrutinized via Agora in order to determine whether they are associated with an increased susceptibility to AD.

### 2.2. Dataset Selection

PubMed was inquired for studies on RNA-sequencing of the nasal mucosa, olfactory bulb, and amygdala samples from COVID-19 patients vs. controls, representing the olfactory pathway from the nasal epithelium to the amygdala, the main processing hub for odor identification. 

For nasal mucosa transcriptomes, the query “nasal mucosa; SARS-CoV-2; gene expression” was submitted to PubMed on 31 December 2023, retrieving 40 studies after excluding preprints. Among these, 39 studies did not provide gene expression data on ciliated cells infected by SARS-CoV-2. Ziegler et al.’s study [28] was selected for further analysis (*n* = 58). 

For amygdala mucosa transcriptomes, the query “amygdala; SARS-CoV-2; gene expression” was submitted to PubMed on 31 December 2023, retrieving two studies after excluding preprints. Serrano et al.’s study [35] was selected for further analysis (*n* = 36; 18 controls vs. 18 COVID-19 patients). 

For olfactory bulb transcriptomes, the query “olfactory bulb; SARS-CoV-2; gene expression” was submitted to PubMed on 31 December 2023, retrieving six studies after excluding preprints. Serrano et al.’s study [35] was selected among them (*n* = 40; 20 controls vs. 20 COVID-19 patients). 

For the Alzheimer’s disease datasets, we selected data from Das and colleagues [34] as it reported on sequencing of spatially resolved data, accounting for gene expression changes in relation to either Aβ plaques or neurofibrillary tangles. This unique dataset would allow us to detect, if present, IFN-I signatures in glia and in association with either pathology [36] and, by comparing them with the COVID-19 IFN-I signatures, determine overlap. 

The search strategy for each COVID-19 dataset is reported in Supplementary Materials File S1. We excluded studies based on the following criteria:

1. Regarding study design:
  - Reviews.
  - Studies on children.
  - In vitro or animal experiments, including gene expression data derived from such experiments.
  - Study design unrelated to COVID-19 or focusing on a specific comorbidity.
  - Sample size <5 per group.
2. Regarding gene expression data:
  - Gene expression data limited on SARS-CoV-2 entry factors.
  - Gene expression data are limited on immune or other gene panels.

The dataset available by Das and colleagues was specifically selected due to its design and, specifically, the user of laser capture microdissection to capture and sequence temporal lobe Aβ plaques from AD patients and controls, followed by RNA sequencing. The data generated by this approach would provide unique insight into whether IFN-I signatures result from a specific aspect of AD neuropathology in the brain, as we hypothesized. 

### 2.3. Leveraging Gene Set Enrichment Analysis (GSEA) Results from COVID-19 and Alzheimer’s Disease Datasets

Gene-set enrichment analysis was performed as a confirmatory procedure for the nasal ciliated cell gene set and an exploratory procedure following the identification of shared genes between the COVID-19 and AD datasets. As a confirmatory procedure, GSEA was used to extract an IFN-I gene from the Ziegler et al. dataset utilizing the Reactome database in order for it to be directly comparable with the other signatures reported from the OB, amygdala, and AD datasets (Supplementary File S2). As an exploratory procedure, GSEA aimed to reveal whether genes shared between COVID-19 and AD datasets enriched innate immune pathways and specifically IFN-I. GSEA was performed via Enrichr using standard parameters [37]. In brief, gene lists (sets) are uploaded to Enrichr, and subsequently, enrichment is determined by several available ranking methods. *p*-values are calculated via Fisher’s exact test, with the hypothesis that input genes are independent, i.e., not a network. The q-value is an adjusted *p*-value calculated by applying the Benjamini-Hochberg method. While the other two methods provided by Enrichr, the odds ratio ranking and combined score were in accordance with the adjusted *p*-values [38], we report on the latter as it is generalizable in IFN-I pathways we obtained directly from referenced datasets.

For all analyses, *p*-values and adjusted *p*-values/q-values < 0.05 were considered statistically significant. 

### 2.4. Detection of Specific Overlapping Genes in Type I Interferon Signatures and Their Determination as Gene Network–the Minimal Dysregulated Network

InteractiVenn [39] was used to identify shared genes across significant type I interferon signatures in COVID-19 transcriptomes (Represented by the Reactome “R-HSA-909733-Interferon alpha/beta signaling”) from the reconstructed transolfactory pathway and the type I interferon signature identified in the Alzheimer’s disease transcriptome used for comparison. These genes were subsequently investigated by STRING [40] in order to determine whether their respective proteins represent a valid interactome. To achieve this, STRING integrates and interrogates multiple sources of data, including text mining, databases of biological experiments, genomic context, and co-expression computational predictions. Following the input of a candidate interactome, STRING reports on a protein–protein Interaction (PPI) score; PPI scores < 0.05 indicate significant enrichment of this network., i.e., significantly more interactions than what would be expected by chance between the members of a random interactor gene set of the same size.

### 2.5. Leveraging the Agora Multi-Omics Database to Identify Gene-Disease Associations with Alzheimer’s Disease

In order to determine associations between interferon-associated genes shared across COVID-19 tissue that were also associated with Alzheimer’s disease, we leveraged the Agora resource. Agora (Available from: https://agora.adknowledgeportal.org/ Accessed 5 January 2024) is a web application that compiles high-dimensional human transcriptomic, proteomic, and metabolomic evidence from multiple sources and provides metrics on gene-disease association with Alzheimer’s disease (AD). The Agora web resource is supported by the National Institute on Aging’s Accelerating Medicines Partnership in Alzheimer’s Disease (AMP-AD) consortium and Target Enablement to Accelerate Therapy Development for Alzheimer’s Disease (TREAT-AD) centers, as well as other collaborating research teams. 

For the intended comparisons, we considered the “RNA–differential expression” and the “AD diagnosis–male and females” via gene comparison tool, and a significance value of <0.05 was considered statistically significant for each tissue anterior cingulate cortex (ACC); Cerebellum (CBE); dorsolateral prefrontal cortex (DLPFC); frontal pole (FP); inferior frontal gyrus (IFG); posterior cingulate cortex (PCC); parahippocampal gyrus (PHG); superior temporal gyrus (STG); temporal cortex (TCX). For each gene input, the tool returns three scores (Target risk score, range 0–5; Genetic Risk score, range 0–3; Multi-omic Risk score, range 0–2; higher scores indicate a greater likelihood of disease association). The Multi-omic Risk summarizes transcriptomic and proteomic evidence supporting the target gene’s association with late-onset Alzheimer’s Disease from multiple studies. The Genetic Risk Score is a summary of genetic evidence supporting the target gene’s association with late-onset Alzheimer’s Disease from multiple genetic studies. Finally, the Target Risk Score (TRS) represents the gene target’s general relevance to Alzheimer’s Disease. The TRS is the sum of the target’s Genetic Risk Score and Multi-omic Risk Score, and the higher the score, the more likely the gene involved in AD pathogenesis and confer risk. In this study, we report on genes with TRS > 2.5 and statistically significant differential expression in at least one CNS area.

## 3. Results

### 3.1. Interferon Signaling Pathways Is Significantly Enriched along the Transolfactory Route in COVID-19 and in Response to Alzheimer’s Disease Pathology

GSEA identified two signatures significantly enriching the R-HSA-909733-“Interferon alpha/beta signaling” pathway for olfactory bulb and amygdala donated by COVID-19 patients n_olfactory bulb_20 genes and n_amygdala_ = 45 genes, correspondingly). In turn, GSEA via Enrichr returned the significantly enriched “Interferon alpha/beta signaling” Reactome pathway (n_ciliated_ = 31 genes) leveraging the Ziegler et al.’s dataset. In GSEA results reported by Das and colleagues, the same pathway (R-HSA-909733; n_AD_ = 73 genes) was significantly enriched in the plaques vs. tangles subset of comparisons.

Correspondingly, gene-set enrichment analysis of genes differentially expressed in ciliated cells from COVID-19 patients revealed that the Reactome pathway R-HSA-909733-“Interferon alpha/beta signaling” was significantly enriched, as originally reported [38]. Genes enriching this pathway for each tissue subset were subsequently used to plot a Venn diagram via InteractiVenn and determine those that were shared between nasal ciliated cells, OB, amygdala, and the AD interferon signatures.

### 3.2. Shared Genes between the Transolfactory Route and in Response to Alzheimer’s Disease Pathology Represent a Type I Interferon Network Containing IFITM and OAS Family Genes

**Figure 1:**
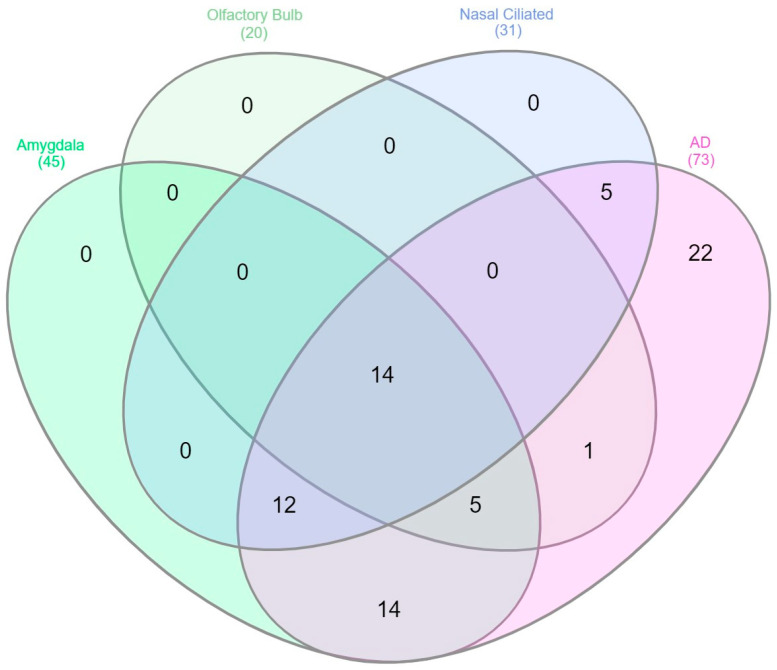
Venn diagram of genes overlapping between all COVID-19 transcriptomes (nasal ciliated cells, olfactory bulb, amygdala) and the Alzheimer’s disease pathology dataset. AD: Alzheimer’s Disease; OB: Olfactory bulb.

**Figure 2:**
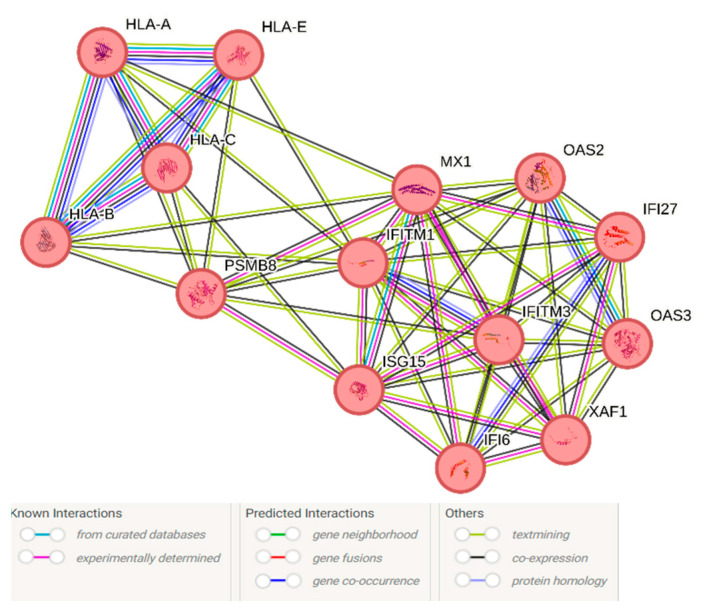
Network view of the 14-gene signature overlapping between COVID-19 tissues and Alzheimer’s disease pathology. Each node represents a protein, whereas edges represent protein–protein interactions. Each line represents the synthesis of several lines of evidence denoting either known or predicted interactions, i.e., from curated databases, experimentally determined gene fusions, gene co-occurrence, text-mining, co-expression, and protein homology. Line thickness repres ents the strength of association (normalized within a 0–1 range). Red corresponds to genes significantly enriching the Type I interferon gene signature.

The InteractiVenn approach revealed all genes enriching the IFN-I signature from the AD study overlapped with at least one COVID-19 tissue. Out of these, 14 genes overlapped between COVID-19 (nasal ciliated cells, olfactory bulb, amygdala) vs. Alzheimer’s disease pathology IFN-I transcriptomes (Figure 1 and Supplementary Files S3 and S4). STRING indicated that these 14 genes corresponded to a significantly enriched PPI network (PPI enrichment *p*-value < 1 × 10^−10^; Figure 2 and Supplementary File S5). Significantly enriched biological pathways and functions associated with this interactome included innate immunity, host–virus interactions, and response to interferon beta and gamma signaling (Table 1 and Supplementary File S6).

### 3.3. Gene-Disease Associations between the Type I Interferon Signature and Alzheimer’s Disease Diagnosis

Scrutinizing the Agora resource, we determined that 9 out of 14 genes had a target risk score >2.5 and were significantly differentially expressed in AD vs. control comparisons, namely HLA-C (4.74), HLA-B (4.68), HLA-A (4.33), PSMB8 (4.05), IFITM3 (3.42), HLA-E (3.17), IFITM1 (3.08), OAS2 (2.92) and MX1 (2.6). See Supplementary File S7 for the raw data. 

## 4. Discussion

Our study supports a growing body of research that indicates a global challenge of type I interferon signaling secondary to SARS-CoV-2 infection. Our findings suggest a continuum of IFN-I dysregulation exists along the transolfactory route of COVID-19 patients regardless of neuroinvasion, extending from the nasal epithelium to the amygdala. The genes implicated are furthermore shown to characterize the glial response to Alzheimer’s disease pathology and are furthermore potentially associated with its diagnosis as determined by aggregated multi-omic evidence. These findings suggest that type I interferon signaling represents a robust candidate for the mechanism of cognitive impairment in the post-COVID spectrum and that it may contribute to glial-driven neuroinflammation in the setting of Alzheimer’s disease via dysregulated IFITM and OAS family genes and impaired cognitive resilience mechanisms.

### 4.1. Type I Interferon as a Mechanism for Cognitive Impairment in Long COVID

Neuroimaging and clinical evidence on the impact of COVID-19 in the CNS converge in neurodegenerative changes affecting areas associated with cognition, as well as the extended olfactory network [27,41,42,43]. While neuroanatomical studies have failed to show productive neuroinfection in the majority of patients [31], cognitive impairment remains a frequent complaint and one that causes significant impairment [44]. Dysregulated interferon signaling secondary to SARS-CoV-2, especially in sites proximal to the CNS, is well documented [29], with the transolfactory route presenting an attractive target of study due to projections to the limbic system. Dysregulations of IFN-I directly impact tonic IFN-I in the CNS, a signaling biorhythm that is necessary for hippocampal synaptic plasticity, adult neo-neurogenesis, and, by extension, cognitive function [6,45]. Our study, therefore, identifies the presence of dysregulated IFN-I cascades in patients with COVID-19, an adequate mechanism by which cognitive impairment and neurodegenerative changes can occur. Our current findings support our proposed model [2] and the concept of indolent neuroinfection restricted proximally to its porting site [46]. 

Notably, independent studies have provided support to our model [4,5,47,48]. Notably, while we focused on a transolfactory route for relaying IFN-I signaling, several studies have found IFN-I dysregulations in sites such as the frontal cortex [30] and brain endothelial cells [20,49,50,51]. Per our expanded model [3], infection beyond the porting site may not be necessary, but it is not precluded: indolent or “slow burning” infection of astrocytes by SARS-CoV-2 has been shown to occur [19,52,53] and may represent yet another mechanism by which aberrant type I interferon signaling impacts cognition and associated neuroanatomical hubs [6]. Most importantly, our current findings suggest dysregulated IFN-I in the CNS following COVID-19, and by extent, impaired plasticity and neurogenesis–effects that have already been observed and retroactively provide firm support to the mechanistic aspect of our findings [6,42,43].

The plausibility of IFN-I-centric crosstalk between central and peripheral immunity is furthermore supported by the robust relationship between individual interactors within the 14-gene signature and innate immune processes relevant to both the periphery and the CNS. Human Leukocyte Antigen (HLA) gene expression is one such example of genes linking peripheral and CNS immune processes with neuronal survival, whose dysfunction has been previously linked to AD [54]. Similarly, the significantly enriched pathway “DAP12 interactions” refers to interactions mediated by the DAP12/TYROBP transmembrane adaptor protein that mediates both peripheral innate immune processes and C1q-mediated synaptotoxicity [55].

A direct implication of this analysis, however, is the implication of a druggable IFN-I network in SARS-CoV-2’s effect on cognition, highlighted by the enrichment of an interferon beta–1a interactome in our findings [56]. Administration of interferon beta has been shown to ameliorate glial neuroinflammation in previous studies of SARS-CoV-2 infected cells [30,57], and further research into nasal delivery of interferon beta, as well as its timing in the disease, may be warranted. 

### 4.2. Type I Interferon Signaling as Common Ground between COVID-19 and Alzheimer’s Disease: A Hint towards Nucleic Acid Immunity and the cGAS-STING-IFITM3 Axis

In previous works, we hypothesized that an overlap between COVID-19 and Alzheimer’s disease was plausible on the premise of shared innate immunity dysregulations, specifically involving IFN-I [2,3,56]. The hippocampus, a site critical for cognition, is reliant upon tight maintenance of tonic IFN-I, with even peripheral immune challenges resulting in synaptic loss and neurodegeneration [6,58,59,60]. 

Taken together, these studies provide further support to crosstalk between tonic IFN-I within the CNS and the periphery. At the same time, IFN-I tonicity within the CNS tightly regulates several homeostatic processes, including adult neo-neurogenesis, synaptic plasticity [6,59,61], and proteostasis (represented by the PSMB8 gene in our findings, a constituent of the immunoproteasome; Table 1). In turn, aberrantly phosphorylated tau [13,62], beta-amyloid, and intrinsic PAMPs [10,63] affect IFN-I tonicity mainly through microglial and endothelial cell activation of IFN-I coupled pathways, such as the DNA-sensing cGAS-STING pathway [64] and the RNA-sensing OAS antiviral pathway [65], also enriched in our analysis (Table 1). Notably, the X-linked inhibitor of apoptosis (XIAP)–associated factor 1 (XAF1) identified herein is an interferon-stimulated gene (such as ISG15, also identified in our study) that acts as a positive regulated of innate immunity and specifically anti-RNA responses [66]; Notably, in deficient nonsense-mediated decay processes and leakage of intrinsic nucleic acids may simulate viral infection by stimulating these nucleic acid surveillance systems and result in microgliosis and IFN-I upregulation, as previously shown [63,67]. 

SARS-CoV-2’s effects on brain organoids, animal models, and neuropathological studies have consistently involved tauopathy, microglial activation, and neuronal loss in the absence of productive infection [52,53,68,69,70,71]. Our hypothesis and the work presented herein support the concept of an outside-in disruption of tonic IFN-I in the CNS as a primer for these events. 

Is this disrupted IFN-I tonicity an important contributor to Alzheimer’s disease pathobiology? A growing body of research posits that infections, particularly viral, increase the risk for Alzheimer’s and related dementias [72]. Aside from our own study [2], others have also compared Alzheimer’s disease and COVID-19 transcriptomes and found shared dysregulations in innate immune pathways, including IFITM and OAS family genes both in neuropathological studies and murine models [18,73]. IFITM3 is of particular note as its expression has been shown to be modulated by cGAS-STING, a pathway that is critical for SARS-CoV-2 infection [74] as it is for resilience against tau [62] and Aβ [75] induced neuroinflammation–with IFITM3 itself being an amyloidogenic gamma secretase modulator [14]. Notably, the cGAS-STING3 axis is responsive to both cytosolic DNA regardless of its origin (i.e., intrinsic or xenobiotic) and to extrinsic tau fibrils [13] and Aβ seeds [75]. Downstream cGAS-STING-mediated activation of the [NACHT, LRR, and PYD domains-containing protein 3 (NLRP3) inflammasome under sterile and non-sterile triggers [76,77], however, may exacerbate both tau and Aβ pathology and its propagation, in part via IFN-I signaling relayed from microglia to neurons [78]. This would imply a potentially self-perpetuating inflammatory loop, whose drivers may be interchangeable: nucleic acids from invading pathogens, nucleic acids from damaged mitochondria, and proteopathic seeds caught in a positive feedback loop with cGAS-STING at its epicenter and IFN-I-driven pathology as an added detriment. 

### 4.3. Limitations, Strengths, and Outstanding Questions

Our study’s findings should be interpreted within the context of its limitations. As this was an in silico investigation, we cannot confirm our results in a prospective manner or via replication of the studies whose datasets were re-analyzed; this can only be performed by designing a similar experiment prospectively. Rather, we aimed to identify IFN-I in gene expression data that had previously been generated. The main idea behind attempting this synthesis and the comparisons described herein is to examine the plausibility of an IFN-I centric model for SARS-CoV-2’s effect on the CNS and its overlap with Alzheimer’s disease as described in previous works from our group [2,17,21]; notably, aside from our work or the transolfactory concept explored herein, independent studies have provided further support to this concept [18,19,20]. 

A major limitation to be considered when interpreting our analysis is the current data source diversity. Specifically, there is a single study on gene expression data from the amygdala and olfactory bulb combined (Supplementary Materials File S1; *n* = 36; 18 controls vs. 18 COVID-19 patients donating amygdala tissue, specifically [35]). Likewise, detailed data on ciliated cells infected by COVID-19 fitting the criteria of our study were available from one out of 40 studies (Supplementary Materials File S1; *n* = 58 [28]). The lack of other studies providing data on the extended olfactory network enforces a modicum of selection bias that is currently unavoidable; therefore, its impact on the reproducibility and the context of our work should be tested by subsequent studies. Indirect evidence (i.e., from studies of human brain interactomes albeit different loci, however, indicates that IFN-I dysregulation is a plausible and, as elsewhere noted, dysregulated mechanism in the CNS following COVID-19 exposure and in the absence of fulminant neuroinfection [30]). Our analyses, despite their limitations on that aspect, would therefore reinforce this concept.

The added value of the current investigation is that COVID-19 transcriptomes are derived from nasal epithelia and the establishment of IFN-I dysregulation along contiguous anatomically and functionally connected sites; subsequently, we aimed to determine the potential association of this derived interactome with IFN-I signatures in Alzheimer’s disease, as previously described in our works [2]. A consequent limitation of our study, however, is that aside from the tissues examined, we cannot infer whether other areas critical for cognition are directly affected by IFN-I. While not analyzed in our current study, frontal lobe transcriptomes from COVID-19 patients have been reported to share IFN-I perturbations with frontal lobe transcriptomes from Alzheimer’s disease patients in at least one study [79]. Others have also reported the choroid plexus as a potential site of inflammation with cognitive consequences, and IFN-I dysregulation here may be a shared feature between COVID-19 and Alzheimer’s disease [5,19,45]. Arguably, however, the regulation of both type I and type II interferon signaling is important for both COVID-19 and Alzheimer’s disease, a point that has not been exhaustively explored herein.

Another limitation and an outstanding research question in the current analysis is it was not designed to assess the contribution of exosomes in the neuropathology caused by COVID-19, which could transfer nucleic acids and cellular debris that could potentially trigger nucleic acid-responsive innate immune mechanisms, such as cGAS-STING and OAS. Gene expression on the BBB [11,80,81] or the choroid plexus [5,19,45] were similarly not examined, albeit, as previously mentioned, both IFN-I dysregulation and IFITM3 networks have been reported. Likewise, other aspects of COVID-19’s trajectory that may implicate innate immunity within the CNS, such as RNAemia [82] in the setting of a disrupted BBB, were not the objective of the current study. 

A final limitation and, similarly, an outstanding research question is the direct generalizability of an IFN-I-centric rather than a pathogen-centric model of cognitive impairment and contribution to Alzheimer’s disease pathology. The production of neurotoxic amyloids has been previously shown to occur in *Pseudomonas Aeruginosa* pneumonia [83,84], and Aβ oligomers have been shown to inhibit and opsonize IAV [85]–providing traction to their potential role as antimicrobial peptides [86]. SARS-CoV-2–*Pseudomonas Aeruginosa* coinfection [87] may favor the latter’s expansion in the nasal epithelia [88], providing another culprit capable of mechanistically inducing beta amyloidosis in the olfactory and lung epithelia [89], and an inducer of innate immune responses, including type I interferon signaling.

## 5. Conclusions

Our study provides further support to a type I interferon signaling dysregulation along the extended olfactory network, ranging from the nasal epithelium and extending to the amygdala. We furthermore identify the 14 genes implicated in this dysregulated pathway with Alzheimer’s disease pathology, among which HLA-C, HLA-B, HLA-A, PSMB8, IFITM3, HLA-E, IFITM1, OAS2, and MX1 as genes with associated conferring increased risk for the latter. The presence of genes associated with the response to IFNβ in this signature, as per our previous works [56] and as shown experimentally in other works [30,57], suggests that further research into this class of therapeutics may be warranted. 

## Figures and Tables

**Table 1:** Significantly enriched Reactome pathways associated with the 14 gene signature.

Reactome Term ID	Term Description	False Discovery Rate
*R-HSA-909733*	Interferon alpha/beta signaling	5.68 × 10^−31^
*R-HSA-877300*	Interferon-gamma signaling	1.51 × 10^−8^
*R-HSA-1236977*	Endosomal/Vacuolar pathway	8.23 × 10^−8^
*R-HSA-1236974*	ER-Phagosome pathway	1.40 × 10^−6^
*R-HSA-983170*	Antigen Presentation: Folding, assembly, and peptide loading of class I MHC	1.84 × 10^−6^
*R-HSA-9705671*	SARS-CoV-2 activates/modulates innate and adaptive immune responses	4.65 × 10^−6^
*R-HSA-198933*	Immunoregulatory interactions between a Lymphoid and a non-Lymphoid cell	5.53 × 10^−6^
*R-HSA-1169410*	Antiviral mechanism by IFN-stimulated genes	5.74 × 10^−5^
*R-HSA-2172127*	DAP12 interactions	0.00049
*R-HSA-1280218*	Adaptive Immune System	0.00097
*R-HSA-5663205*	Infectious disease	0.0027
*R-HSA-8983711*	OAS antiviral response	0.0029
*R-HSA-168249*	Innate Immune System	0.005

## Data Availability

Data are available upon request and publicly.

## Supplementary Materials

The following supporting information can be downloaded at: https://www.mdpi.com/article/10.3390/cimb46050277/s1.

## References

[1] de Erausquin G.A., Snyder H., Carrillo M., Hosseini A.A., Brugha T.S., Seshadri S. (2021). The chronic neuropsychiatric sequelae of COVID-19: The need for a prospective study of viral impact on brain functioning. Alzheimer’s Dement..

[2] Vavougios G.D., Nday C., Pelidou S.H., Gourgoulianis K.I., Stamoulis G., Doskas T., Zarogiannis S.G. (2021). Outside-in induction of the IFITM3 trafficking system by infections, including SARS-CoV-2, in the pathobiology of Alzheimer’s disease. Brain Behav. Immun. Health.

[3] Vavougios G.D., de Erausquin G.A., Snyder H.M. (2022). Type I interferon signaling in SARS-CoV-2 associated neurocognitive disorder (SAND): Mapping host-virus interactions to an etiopathogenesis. Front. Neurol..

[4] Tan P.H., Ji J., Hsing C.H., Tan R., Ji R.R. (2022). Emerging Roles of Type-I Interferons in Neuroinflammation, Neurological Diseases, and Long-Haul COVID. Int. J. Mol. Sci..

[5] Suzzi S., Tsitsou-Kampeli A., Schwartz M. (2023). The type I interferon antiviral response in the choroid plexus and the cognitive risk in COVID-19. Nat. Immunol..

[6] Hosseini S., Michaelsen-Preusse K., Grigoryan G., Chhatbar C., Kalinke U., Korte M. (2020). Type I Interferon Receptor Signaling in Astrocytes Regulates Hippocampal Synaptic Plasticity and Cognitive Function of the Healthy CNS. Cell Rep..

[7] Ekdahl C.T., Claasen J.-H., Bonde S., Kokaia Z., Lindvall O. (2003). Inflammation is detrimental for neurogenesis in adult brain. Proc. Natl. Acad. Sci. USA.

[8] Kim H.S., Shin S.M., Kim S., Nam Y., Yoo A., Moon M. (2022). Relationship between adult subventricular neurogenesis and Alzheimer’s disease: Pathologic roles and therapeutic implications. Front. Aging Neurosci..

[9] Roy E., Cao W. (2022). Glial interference: Impact of type I interferon in neurodegenerative diseases. Mol. Neurodegener..

[10] Roy E.R., Wang B., Wan Y.-w., Chiu G., Cole A., Yin Z., Propson N.E., Xu Y., Jankowsky J.L., Liu Z. (2020). Type I interferon response drives neuroinflammation and synapse loss in Alzheimer disease. J. Clin. Investig..

[11] Jana A., Wang X., Leasure J.W., Magana L., Wang L., Kim Y.-M., Dodiya H., Toth P.T., Sisodia S.S., Rehman J. (2022). Increased Type I interferon signaling and brain endothelial barrier dysfunction in an experimental model of Alzheimer’s disease. Sci. Rep..

[12] Duarte N., Shafi A.M., Penha-Gonçalves C., Pais T.F. (2023). Endothelial type I interferon response and brain diseases: Identifying STING as a therapeutic target. Front. Cell Dev. Biol..

[13] Udeochu J.C., Amin S., Huang Y., Fan L., Torres E.R.S., Carling G.K., Liu B., McGurran H., Coronas-Samano G., Kauwe G. (2023). Tau activation of microglial cGAS–IFN reduces MEF2C-mediated cognitive resilience. Nat. Neurosci..

[14] Hur J.Y., Frost G.R., Wu X., Crump C., Pan S.J., Wong E., Barros M., Li T., Nie P., Zhai Y. (2020). The innate immunity protein IFITM3 modulates γ-secretase in Alzheimer’s disease. Nature.

[15] Prelli Bozzo C., Nchioua R., Volcic M., Koepke L., Krüger J., Schütz D., Heller S., Stürzel C.M., Kmiec D., Conzelmann C. (2021). IFITM proteins promote SARS-CoV-2 infection and are targets for virus inhibition in vitro. Nat. Commun..

[16] Shi G., Kenney A.D., Kudryashova E., Zani A., Zhang L., Lai K.K., Hall-Stoodley L., Robinson R.T., Kudryashov D.S., Compton A.A. (2021). Opposing activities of IFITM proteins in SARS-CoV-2 infection. EMBO J..

[17] Vavougios G.D., Breza M., Mavridis T., Krogfelt K.A. (2021). FYN, SARS-CoV-2, and IFITM3 in the neurobiology of Alzheimer’s disease. Brain Disord..

[18] Magusali N., Graham A.C., Piers T.M., Panichnantakul P., Yaman U., Shoai M., Reynolds R.H., Botia J.A., Brookes K.J., Guetta-Baranes T. (2021). Genetic variability associated with *OAS1* expression in myeloid cells increases the risk of Alzheimer’s disease and severe COVID-19 outcomes. bioRxiv.

[19] Yang A.C., Kern F., Losada P.M., Agam M.R., Maat C.A., Schmartz G.P., Fehlmann T., Stein J.A., Schaum N., Lee D.P. (2021). Dysregulation of brain and choroid plexus cell types in severe COVID-19. Nature.

[20] Zhou Y., Xu J., Hou Y., Leverenz J.B., Kallianpur A., Mehra R., Liu Y., Yu H., Pieper A.A., Jehi L. (2021). Network medicine links SARS-CoV-2/COVID-19 infection to brain microvascular injury and neuroinflammation in dementia-like cognitive impairment. Alzheimer’s Res. Ther..

[21] Vavougios G.D., Nday C., Pelidou S.H., Zarogiannis S.G., Gourgoulianis K.I., Stamoulis G., Doskas T. (2020). Double hit viral parasitism, polymicrobial CNS residency and perturbed proteostasis in Alzheimer’s disease: A data driven, in silico analysis of gene expression data. Mol. Immunol..

[22] Daroische R., Hemminghyth M.S., Eilertsen T.H., Breitve M.H., Chwiszczuk L.J. (2021). Cognitive Impairment After COVID-19—A Review on Objective Test Data. Front. Neurol..

[23] Pirker-Kees A., Platho-Elwischger K., Hafner S., Redlich K., Baumgartner C. (2021). Hyposmia Is Associated with Reduced Cognitive Function in COVID-19: First Preliminary Results. Dement. Geriatr. Cogn. Disord..

[24] Baek M.S., Cho H., Lee H.S., Lee J.H., Ryu Y.H., Lyoo C.H. (2020). Effect of A/T/N imaging biomarkers on impaired odor identification in Alzheimer’s disease. Sci. Rep..

[25] Lafaille-Magnan M.-E., Poirier J., Etienne P., Tremblay-Mercier J., Frenette J., Rosa-Neto P., Breitner J.C.S., For the PREVENT-AD Research Group (2017). Odor identification as a biomarker of preclinical AD in older adults at risk. Neurology.

[26] de Erausquin G.A., Snyder H., Brugha T.S., Seshadri S., Carrillo M., Sagar R., Huang Y., Newton C., Tartaglia C., Teunissen C. (2022). Chronic neuropsychiatric sequelae of SARS-CoV-2: Protocol and methods from the Alzheimer’s Association Global Consortium. Alzheimer’s Dement..

[27] Douaud G., Lee S., Alfaro-Almagro F., Arthofer C., Wang C., McCarthy P., Lange F., Andersson J.L.R., Griffanti L., Duff E. (2022). SARS-CoV-2 is associated with changes in brain structure in UK Biobank. Nature.

[28] Ziegler C.G.K., Miao V.N., Owings A.H., Navia A.W., Tang Y., Bromley J.D., Lotfy P., Sloan M., Laird H., Williams H.B. (2021). Impaired local intrinsic immunity to SARS-CoV-2 infection in severe COVID-19. Cell.

[29] Hatton C.F., Botting R.A., Dueñas M.E., Haq I.J., Verdon B., Thompson B.J., Spegarova J.S., Gothe F., Stephenson E., Gardner A.I. (2021). Delayed induction of type I and III interferons mediates nasal epithelial cell permissiveness to SARS-CoV-2. Nat. Commun..

[30] Mavrikaki M., Lee J.D., Solomon I.H., Slack F.J. (2022). Severe COVID-19 is associated with molecular signatures of aging in the human brain. Nature Aging.

[31] Meinhardt J., Radke J., Dittmayer C., Franz J., Thomas C., Mothes R., Laue M., Schneider J., Brünink S., Greuel S. (2021). Olfactory transmucosal SARS-CoV-2 invasion as a port of central nervous system entry in individuals with COVID-19. Nat. Neurosci..

[32] Schreiber G. (2020). The Role of Type I Interferons in the Pathogenesis and Treatment of COVID-19. Front. Immunol..

[33] Ahn J.H., Kim J., Hong S.P., Choi S.Y., Yang M.J., Ju Y.S., Kim Y.T., Kim H.M., Rahman M.D.T., Chung M.K. (2021). Nasal ciliated cells are primary targets for SARS-CoV-2 replication in the early stage of COVID-19. J. Clin. Investig..

[34] Das S., Li Z., Wachter A., Alla S., Noori A., Abdourahman A., Tamm J.A., Woodbury M.E., Talanian R.V., Biber K. (2024). Distinct transcriptomic responses to Aβ plaques, neurofibrillary tangles, and APOE in Alzheimer’s disease. Alzheimer’s Dement..

[35] Serrano G.E., Walker J.E., Tremblay C., Piras I.S., Huentelman M.J., Belden C.M., Goldfarb D., Shprecher D., Atri A., Adler C.H. (2022). SARS-CoV-2 Brain Regional Detection, Histopathology, Gene Expression, and Immunomodulatory Changes in Decedents with COVID-19. J. Neuropathol. Exp. Neurol..

[36] Mathys H., Adaikkan C., Gao F., Young J.Z., Manet E., Hemberg M., De Jager P.L., Ransohoff R.M., Regev A., Tsai L.H. (2017). Temporal Tracking of Microglia Activation in Neurodegeneration at Single-Cell Resolution. Cell Rep..

[37] Kuleshov M.V., Jones M.R., Rouillard A.D., Fernandez N.F., Duan Q., Wang Z., Koplev S., Jenkins S.L., Jagodnik K.M., Lachmann A. (2016). Enrichr: A comprehensive gene set enrichment analysis web server 2016 update. Nucleic Acids Res..

[38] Xie Z., Bailey A., Kuleshov M.V., Clarke D.J.B., Evangelista J.E., Jenkins S.L., Lachmann A., Wojciechowicz M.L., Kropiwnicki E., Jagodnik K.M. (2021). Gene Set Knowledge Discovery with Enrichr. Curr. Protoc..

[39] Heberle H., Meirelles G.V., da Silva F.R., Telles G.P., Minghim R. (2015). InteractiVenn: A web-based tool for the analysis of sets through Venn diagrams. BMC Bioinform..

[40] Szklarczyk D., Gable A.L., Nastou K.C., Lyon D., Kirsch R., Pyysalo S., Doncheva N.T., Legeay M., Fang T., Bork P. (2021). The STRING database in 2021: Customizable protein-protein networks, and functional characterization of user-uploaded gene/measurement sets. Nucleic Acids Res..

[41] Crook H., Ramirez A., Hosseini A., Vavougyios G., Lehmann C., Bruchfeld J., Schneider A., D’Avossa G., Lo Re V., Salmoiraghi A. (2023). European Working Group on SARS-CoV-2: Current Understanding, Unknowns, and Recommendations on the Neurological Complications of COVID-19. Brain Connect..

[42] Bayat A.H., Azimi H., Hassani Moghaddam M., Ebrahimi V., Fathi M., Vakili K., Mahmoudiasl G.R., Forouzesh M., Boroujeni M.E., Nariman Z. (2022). COVID-19 causes neuronal degeneration and reduces neurogenesis in human hippocampus. Apoptosis.

[43] Muccioli L., Sighinolfi G., Mitolo M., Ferri L., Jane Rochat M., Pensato U., Taruffi L., Testa C., Masullo M., Cortelli P. (2023). Cognitive and functional connectivity impairment in post-COVID-19 olfactory dysfunction. Neuroimage Clin..

[44] Deer R.R., Rock M.A., Vasilevsky N., Carmody L., Rando H., Anzalone A.J., Basson M.D., Bennett T.D., Bergquist T., Boudreau E.A. (2021). Characterizing Long COVID: Deep Phenotype of a Complex Condition. EBioMedicine.

[45] Baruch K., Deczkowska A., David E., Castellano J.M., Miller O., Kertser A., Berkutzki T., Barnett-Itzhaki Z., Bezalel D., Wyss-Coray T. (2014). Aging. Aging-induced type I interferon response at the choroid plexus negatively affects brain function. Science.

[46] Vavougios G.D. (2020). Potentially irreversible olfactory and gustatory impairments in COVID-19: Indolent vs. fulminant SARS-CoV-2 neuroinfection. Brain Behav. Immun..

[47] Lee J.S., Shin E.-C. (2020). The type I interferon response in COVID-19: Implications for treatment. Nat. Rev. Immunol..

[48] Wu B., Ramaiah A., Garcia G., Hasiakos S., Arumugaswami V., Srikanth S. (2022). ORAI1 Limits SARS-CoV-2 Infection by Regulating Tonic Type I IFN Signaling. J. Immunol..

[49] Yang R.-C., Huang K., Zhang H.-P., Li L., Zhang Y.-F., Tan C., Chen H.-C., Jin M.-L., Wang X.-R. (2022). SARS-CoV-2 productively infects human brain microvascular endothelial cells. J. Neuroinflammation.

[50] Wenzel J., Lampe J., Müller-Fielitz H., Schuster R., Zille M., Müller K., Krohn M., Körbelin J., Zhang L., Özorhan Ü. (2021). The SARS-CoV-2 main protease Mpro causes microvascular brain pathology by cleaving NEMO in brain endothelial cells. Nat. Neurosci..

[51] Vavougios G.D., Zarogiannis S.G., Hadjigeorgiou G., Krogfelt K.A., Gourgoulianis K.I. (2022). SARS-CoV-2 and type I interferon signaling in brain endothelial cells: Blurring the lines between friend or foe. Stem Cell Rep..

[52] Kong W., Montano M., Corley M.J., Helmy E., Kobayashi H., Kinisu M., Suryawanshi R., Luo X., Royer L.A., Roan N.R. (2022). Neuropilin-1 Mediates SARS-CoV-2 Infection of Astrocytes in Brain Organoids, Inducing Inflammation Leading to Dysfunction and Death of Neurons. mBio.

[53] Kong D., Park K.H., Kim D.-H., Kim N.G., Lee S.-E., Shin N., Kook M.G., Kim Y.B., Kang K.-S. (2023). Cortical-blood vessel assembloids exhibit Alzheimer’s disease phenotypes by activating glia after SARS-CoV-2 infection. Cell Death Discov..

[54] Wang Z.X., Wan Q., Xing A. (2020). HLA in Alzheimer’s Disease: Genetic Association and Possible Pathogenic Roles. Neuromolecular Med..

[55] Haure-Mirande J.-V., Audrain M., Ehrlich M.E., Gandy S. (2022). Microglial TYROBP/DAP12 in Alzheimer’s disease: Transduction of physiological and pathological signals across TREM2. Mol. Neurodegener..

[56] Vavougios G.D., Mavridis T., Artemiadis A., Krogfelt K.A., Hadjigeorgiou G. (2022). Trained immunity in viral infections, Alzheimer’s disease and multiple sclerosis: A convergence in type I interferon signalling and IFNβ-1a. Biochim. Biophys. Acta Mol. Basis Dis..

[57] Magusali N., Graham A.C., Piers T.M., Panichnantakul P., Yaman U., Shoai M., Reynolds R.H., Botia J.A., Brookes K.J., Guetta-Baranes T. (2021). A genetic link between risk for Alzheimer’s disease and severe COVID-19 outcomes via the OAS1 gene. Brain.

[58] Michalovicz L.T., Lally B., Konat G.W. (2015). Peripheral challenge with a viral mimic upregulates expression of the complement genes in the hippocampus. J. Neuroimmunol..

[59] Petrisko T.J., Bloemer J., Pinky P.D., Srinivas S., Heslin R.T., Du Y., Setti S.E., Hong H., Suppiramaniam V., Konat G.W. (2020). Neuronal CXCL10/CXCR3 Axis Mediates the Induction of Cerebral Hyperexcitability by Peripheral Viral Challenge. Front. Neurosci..

[60] Grabrucker S., Marizzoni M., Silajdžić E., Lopizzo N., Mombelli E., Nicolas S., Dohm-Hansen S., Scassellati C., Moretti D.V., Rosa M. (2023). Microbiota from Alzheimer’s patients induce deficits in cognition and hippocampal neurogenesis. Brain.

[61] Viengkhou B., Hofer M.J. (2023). Breaking down the cellular responses to type I interferon neurotoxicity in the brain. Front. Immunol..

[62] Jin M., Shiwaku H., Tanaka H., Obita T., Ohuchi S., Yoshioka Y., Jin X., Kondo K., Fujita K., Homma H. (2021). Tau activates microglia via the PQBP1-cGAS-STING pathway to promote brain inflammation. Nat. Commun..

[63] Roy E.R., Chiu G., Li S., Propson N.E., Kanchi R., Wang B., Coarfa C., Zheng H., Cao W. (2022). Concerted type I interferon signaling in microglia and neural cells promotes memory impairment associated with amyloid β plaques. Immunity.

[64] Yu L., Liu P. (2021). Cytosolic DNA sensing by cGAS: Regulation, function, and human diseases. Signal Transduct. Target. Ther..

[65] Choi U.Y., Kang J.-S., Hwang Y.S., Kim Y.-J. (2015). Oligoadenylate synthase-like (OASL) proteins: Dual functions and associations with diseases. Exp. Mol. Med..

[66] Kuang M., Zhao Y., Yu H., Li S., Liu T., Chen L., Chen J., Luo Y., Guo X., Wei X. (2023). XAF1 promotes anti-RNA virus immune responses by regulating chromatin accessibility. Sci. Adv..

[67] Sanford S.A.I., Miller L.V.C., Vaysburd M., Keeling S., Tuck B.J., Clark J., Neumann M., Syanda V., James L.C., McEwan W.A. (2024). The type-I interferon response potentiates seeded tau aggregation and exacerbates tau pathology. Alzheimer’s Dement..

[68] Di Primio C., Quaranta P., Mignanelli M., Siano G., Bimbati M., Scarlatti A., Piazza C.R., Spezia P.G., Perrera P., Basolo F. (2023). Severe acute respiratory syndrome coronavirus 2 infection leads to Tau pathological signature in neurons. PNAS Nexus.

[69] Ramani A., Muller L., Ostermann P.N., Gabriel E., Abida-Islam P., Muller-Schiffmann A., Mariappan A., Goureau O., Gruell H., Walker A. (2020). SARS-CoV-2 targets neurons of 3D human brain organoids. EMBO J..

[70] Käufer C., Schreiber C.S., Hartke A.S., Denden I., Stanelle-Bertram S., Beck S., Kouassi N.M., Beythien G., Becker K., Schreiner T. (2022). Microgliosis and neuronal proteinopathy in brain persist beyond viral clearance in SARS-CoV-2 hamster model. EBioMedicine.

[71] Hou Y., Li C., Yoon C., Leung O.W., You S., Cui X., Chan J.F., Pei D., Cheung H.H., Chu H. (2022). Enhanced replication of SARS-CoV-2 Omicron BA.2 in human forebrain and midbrain organoids. Signal Transduct. Target Ther..

[72] Levine K.S., Leonard H.L., Blauwendraat C., Iwaki H., Johnson N., Bandres-Ciga S., Ferrucci L., Faghri F., Singleton A.B., Nalls M.A. (2023). Virus exposure and neurodegenerative disease risk across national biobanks. Neuron.

[73] Green R., Mayilsamy K., McGill A.R., Martinez T.E., Chandran B., Blair L.J., Bickford P.C., Mohapatra S.S., Mohapatra S. (2022). SARS-CoV-2 infection increases the gene expression profile for Alzheimer’s disease risk. Mol. Ther.-Methods Clin. Dev..

[74] Domizio J.D., Gulen M.F., Saidoune F., Thacker V.V., Yatim A., Sharma K., Nass T., Guenova E., Schaller M., Conrad C. (2022). The cGAS–STING pathway drives type I IFN immunopathology in COVID-19. Nature.

[75] Wu Z., Tang W., Ibrahim F., Chen X., Yan H., Tao C., Wang Z., Guo Y., Fu Y., Wang Q. (2023). Aβ Induces Neuroinflammation and Microglial M1 Polarization via cGAS-STING-IFITM3 Signaling Pathway in BV-2 Cells. Neurochem. Res..

[76] Gaidt M.M., Ebert T.S., Chauhan D., Ramshorn K., Pinci F., Zuber S., O’Duill F., Schmid-Burgk J.L., Hoss F., Buhmann R. (2017). The DNA Inflammasome in Human Myeloid Cells Is Initiated by a STING-Cell Death Program Upstream of NLRP3. Cell.

[77] Wang W., Hu D., Wu C., Feng Y., Li A., Liu W., Wang Y., Chen K., Tian M., Xiao F. (2020). STING promotes NLRP3 localization in ER and facilitates NLRP3 deubiquitination to activate the inflammasome upon HSV-1 infection. PLoS Pathog..

[78] Stancu I.-C., Cremers N., Vanrusselt H., Couturier J., Vanoosthuyse A., Kessels S., Lodder C., Brône B., Huaux F., Octave J.-N. (2019). Aggregated Tau activates NLRP3–ASC inflammasome exacerbating exogenously seeded and non-exogenously seeded Tau pathology in vivo. Acta Neuropathol..

[79] Reiken S., Sittenfeld L., Dridi H., Liu Y., Liu X., Marks A.R. (2022). Alzheimer’s-like signaling in brains of COVID-19 patients. Alzheimers’s Dement..

[80] Zhang L., Zhou L., Bao L., Liu J., Zhu H., Lv Q., Liu R., Chen W., Tong W., Wei Q. (2021). SARS-CoV-2 crosses the blood–brain barrier accompanied with basement membrane disruption without tight junctions alteration. Signal Transduct. Target. Ther..

[81] Krasemann S., Haferkamp U., Pfefferle S., Woo M.S., Heinrich F., Schweizer M., Appelt-Menzel A., Cubukova A., Barenberg J., Leu J. (2022). The blood-brain barrier is dysregulated in COVID-19 and serves as a CNS entry route for SARS-CoV-2. Stem Cell Rep..

[82] Tang K., Wu L., Luo Y., Gong B. (2021). Quantitative assessment of SARS-CoV-2 RNAemia and outcome in patients with coronavirus disease 2019. J. Med. Virol..

[83] Scott A.M., Jager A.C., Gwin M., Voth S., Balczon R., Stevens T., Lin M.T. (2020). Pneumonia-induced endothelial amyloids reduce dendritic spine density in brain neurons. Sci. Rep..

[84] Lin M.T., Balczon R., Pittet J.F., Wagener B.M., Moser S.A., Morrow K.A., Voth S., Francis C.M., Leavesley S., Bell J. (2018). Nosocomial Pneumonia Elicits an Endothelial Proteinopathy: Evidence for a Source of Neurotoxic Amyloids in Critically Ill Patients. Am. J. Respir. Crit. Care Med..

[85] White M.R., Kandel R., Tripathi S., Condon D., Qi L., Taubenberger J., Hartshorn K.L. (2014). Alzheimer’s associated β-amyloid protein inhibits influenza A virus and modulates viral interactions with phagocytes. PLoS ONE.

[86] Pastore A., Raimondi F., Rajendran L., Temussi P.A. (2020). Why does the Aβ peptide of Alzheimer share structural similarity with antimicrobial peptides?. Commun. Biol..

[87] Shafran N., Shafran I., Ben-Zvi H., Sofer S., Sheena L., Krause I., Shlomai A., Goldberg E., Sklan E.H. (2021). Secondary bacterial infection in COVID-19 patients is a stronger predictor for death compared to influenza patients. Sci. Rep..

[88] Rhoades N.S., Pinski A.N., Monsibais A.N., Jankeel A., Doratt B.M., Cinco I.R., Ibraim I., Messaoudi I. (2021). Acute SARS-CoV-2 infection is associated with an increased abundance of bacterial pathogens, including Pseudomonas aeruginosa in the nose. Cell Rep..

[89] Voth S., Gwin M., Francis C.M., Balczon R., Frank D.W., Pittet J.-F., Wagener B.M., Moser S.A., Alexeyev M., Housley N. (2020). Virulent Pseudomonas aeruginosa infection converts antimicrobial amyloids into cytotoxic prions. FASEB J..

